# Effects of Low-Dose Antibiotics on Gut Immunity and Antibiotic Resistomes in Weaned Piglets

**DOI:** 10.3389/fimmu.2020.00903

**Published:** 2020-06-10

**Authors:** Qi Hu, Cong Liu, Du Zhang, Ru Wang, Linlin Qin, Qin Xu, Lianqiang Che, Fei Gao

**Affiliations:** ^1^Lingnan Guangdong Laboratory of Modern Agriculture, Genome Analysis Laboratory of the Ministry of Agriculture, Agricultural Genomics Institute at Shenzhen, Chinese Academy of Agricultural Sciences, Shenzhen, China; ^2^Animal Nutrition Institute, Sichuan Agricultural University, Ya'an, China; ^3^Comparative Pediatrics and Nutrition, Department of Veterinary and Animal Sciences, Faculty of Health and Medical Sciences, University of Copenhagen, Frederiksberg, Denmark

**Keywords:** low-dose antibiotics, antimicrobial resistance genes, mobile genetic elements, immunity, intestine, weaned piglet

## Abstract

Widespread antibiotic use increases the risk of livestock acting as potential reservoirs of antimicrobial resistance genes (ARGs) that may be transferred to human and animal pathogens. Particularly, maternal-infant transmission of antibiotics via breastmilk represents a great concern regarding infant health. In this study, we investigated the effects of 4-week low-dose antibiotic (LDA) treatment on the host immunity and antibiotic resistomes in weaned piglets. Transcriptomic analyses of ileum tissues revealed that the affected genes were largely enriched in innate immunity-related pathways. Significantly reduced protein expression of inflammatory factors, i.e., *IFN-*γ*, IL-6* were observed. In addition, analyses of antibiotic resistomes identified a total of 1,021 ARGs related to 39 classes of antibiotics. The samples exhibited highly individual-specific diversity and no significant difference in the structure and diversity of ARGs and mobile gene elements (MGE) after LDA exposure for both colon and ileum samples. Despite of that, there were significant changes in the abundance of two transferrable ARGs [Erm(T) and tcr3] related to the antibiotics administered, implying an increased risk of transferrable antibiotic resistance. There was a significant change in the abundance of one pathogenic species after LDA exposure in the colon samples and one in the ileum samples, but there were no significant differences in the matched ARGs. Collectively, our findings reveal considerable changes in intestinal immunity-related genes, but minimal effects on gut antibiotic resistomes (ARGs and MGEs) in weaned piglets after 4 weeks LDA exposure. Our study provides a foundation for evaluating the longer-term cumulative effects of LDA use, especially the effects of maternal–infant LDA transmission, on antibiotic resistance and risks to infant health.

## Introduction

In-feed antibiotics have been widely used in livestock production to prevent and treat infectious diseases and to control the gut microbiota (1, 2). Non-therapeutic low-dose antibiotics (LDA) are commonly used as feed additives for piglets during the difficult weaning transition period to increase growth, promote gut health, and improve feed efficiency (3). However, antimicrobial resistance is a worldwide public health concern and multidrug antibiotic resistance is partly related to excessive use of antibiotics for livestock. Food-producing animals may act as potential reservoirs of antimicrobial resistance genes (ARGs) that are transferred to and survive in their gut microbiota and then are transferred to human or animal pathogens that enter the gut via the food chain or the environment (4). Previous studies reported antibiotic residue in dairy products (5) and breastmilk (6), regardless of whether the mothers received antibiotics during pregnancy or lactation. Transmission of LDA residue to infants' guts may represent a risk factor for poor infant health. It is important to understand the potential negative side effects of LDA, especially as this preventive antibiotic-related approach is currently being banned in many countries around the world.

The complex and dense microbial environment of the gut is an important natural reservoir of ARGs, and it is also important for the development of the host immune system, thereby influencing infection by intestinal pathogens and the development of allergic and inflammatory bowel diseases (7–11). Metagenomics analyses have indicated the presence of ARGs in the microbiota of human and animal fecal samples and the correlation between the fecal antibiotic resistome and the microbiome (12–14). Further, due to the interactions between microbiota and the host immune system, the antibiotic-mediated alterations to the microbiota composition can result in different immunological consequences. For example, with the antibiotics intake, antibiotics also reduced production of interferon (*IFN*)*-*γ and interleukin (*IL*)*-17* cytokines by CD4+ T lymphocytes in the small intestine by perturbing microbial communities, which would affect the development and differentiation of immune system (9, 15). However, there are few studies on the microbiome-immunity-resistome interactions based on samples from biologically important sites along the gastrointestinal tract, such as the small intestine and colon. The resistome-microbiome-immunity interactions may differ between different gastrointestinal sites due to their widely differing conditions (e.g., luminal pH, metabolites, endogenous antimicrobial peptides and other secretions, gut motility, and food passage).

In a previous study (16), we established a LDA-treated piglet model and demonstrated that 4 weeks of LDA exposure induced clear growth promotion and significantly reduced total bacterial load in the ileum content, but not the colon content. In the present study, we further performed serum immonological analyses and ileum transcriptome analyses to study whether the same LDA regimen influences the systemic or gut immunity. The expression levels of immune-related proteins of ileum tissues were also validated. In addition, we applied deep metagenomic sequencing on samples of ileum and colon contents to investigate the antibiotic resistomes, including ARGs and mobile gene elements (MGEs).

## Materials and Methods

### Animal Treatments and Ileum and Colon Sample Collection

The study was approved by the Sichuan Agriculture University animal welfare committee and carried out in accordance with the National Research Council's Guide for the Care and Use of Laboratory Animals. Twenty-four 21-day-old female weaned piglets were supplied by Sichuan Agricultural University, with a mean body weight of 6.50 ± 0.20 kg. They were randomly divided into two groups, which were fed a basic diet (*n* = 13) or a diet supplemented with LDA (*n* = 11). The LDA regimen involved a premix of the chlortetracycline (10% purity) and the virginiamycin (50% purity) at 750 and 50 mg/kg of feed, respectively. The feed consumed by the piglets was recorded daily and the growth of each piglet was measured weekly. After 4 weeks of LDA exposure, the piglets were euthanized, ileum tissues, and contents of the ileum and colon of each piglet were obtained and flash frozen in liquid nitrogen.

### Blood Sample Collection and Immunological Analysis

Eight milliliter of blood samples were placed in heparinized tubes, followed by centrifugation at 3,000 × g and 4°C for 15 min and then removal and immediate storage of the plasma at −80°C for later analysis. The parameters of routine blood test were analyzed using an automatic blood analyzer (Advia 120, Bayer HealthCare, Tarrytown, NY, USA). Moreover, plasma concentrations of complement 3 (C3), C-Reactive Protein (C-RP), immunoglobulin G (IgG), and immunoglobulin M (IgM) were measured by an automatic biochemical analyzer (Model 7020, Hitachi, Tokyo, Japan) through corresponding commercial kits (Sichuan Maker Biotechnology Inc., Chengdu, China). There was <5% variation between intra-assay and inter-assay coefficients for each assay. Each plasma sample was analyzed in duplicate.

### Transcriptomics Analyses of Ileum Tissues

Total RNA was extracted from the frozen ileum tissue samples (~100 mg each) using Trizol Reagent (Invitrogen, Carlsbad, CA, USA) according to the manufacturer's instructions. The integrity and quality of the RNA were determined by agarose gel electrophoresis (1%) and an assessment of the absorbance (A260/A280). RNA purity was checked using the NanoPhotometer^®^ spectrophotometer (IMPLEN, CA, USA). RNA concentration was measured using Qubit^®^ RNA Assay Kit in Qubit^®^ 2.0 Flurometer (Life Technologies, CA, USA). RNA integrity was assessed using the RNA Nano 6000 Assay Kit of the Agilent Bioanalyzer 2100 system (Agilent Technologies, CA, USA). Sequencing libraries were generated using NEBNext^®^ UltraTM Directional RNA Library Prep Kit for Illumina^®^ (NEB, USA) following the manufacturer's recommendations. Briefly, mRNA was purified from total RNA using poly-T oligo-attached magnetic beads. Fragmentation was carried out using divalent cations under elevated temperature in NEBNext First Strand Synthesis Reaction Buffer (5X). First-strand cDNA was synthesized using random hexamer primer and M-MuLV Reverse Transcriptase (RNaseH-). Second strand cDNA synthesis was subsequently performed using DNA Polymerase I and RNase H. In the reaction buffer, dNTPs with dTTP were replaced by dUTP. Remaining overhangs were converted into blunt ends via exonuclease/polymerase activities. After the adenylation of 3′ ends of DNA fragments, NEBNext Adaptor with a hairpin loop structure was ligated to prepare for hybridization. In order to select cDNA fragments with the right length, the library fragments were purified with AMPure XP system (Beckman Coulter, Beverly, USA). Then 3 μl USER Enzyme (NEB, USA) was used with size-selected, adaptor-ligated cDNA at 37°C for 15 min followed by 5 min at 95°C before PCR. Then PCR was performed with Phusion High-Fidelity DNA polymerase, Universal PCR primers and Index (X) Primer. At last, products were purified (AMPure XP system) and library quality was assessed on the Agilent Bioanalyzer 2100 system. From these libraries, 150-bp paired-end and strand-specific sequence reads were produced with Illumina HiSeq 4000. Hisat2 (v 2.1.0) was employed for performing read mapping to Sus scrofa genome reference (v11.1) (17). Gene expression profiling was based on the number of reads. FPKM (fragments per kb of exon model per million mapped reads) values were used to estimate the expressed values and transcript levels. DEGs were obtained with an *P*-value cutoff < = 0.05 and an absolute fold-change of >= 1.5. KEGG (Kyoto Encyclopedia of Genes and Genome) is the main public database related to the pathway, and it contained 20 pathways related to the immune system. The DEGs related to the immune pathways were identified. KEGG functional enrichment analysis was performed on the clusterProfile R package.

### Enzyme-Linked Immunosorbent Assay (ELISA)

The concentrations of 10 cytokines, including Claudin (*CLDN*)*-1*, interferon (*IFN*)*-*γ, interleukin (*IL*)*-22, IL-6*, myeloid differentiation factor *(MYD)88*, nuclear factor (*NF*)*-*κ*B*, transforming growth factor *(TGF)-*β, toll-like receptor (*TLR*)*4, TLR9*, and TNF receptor associated factor (*TRAF*)*6*, related to innate immune responses were quantified by ELISA (Jianglai Industrial Company, Shanghai, China) according to the manufacturer's instructions.

### DNA Extraction and Shotgun Metagenomic Sequencing

DNA was extracted from the ileum and colon content using a cetyltrimethylammonium bromide (CTAB)/sodium dodecyl sulfate (SDS) method. DNA concentrations were measured using a Qubit^®^ 3.0 Fluorometer (Life Technologies, ThermoFisher Scientific, USA) and DNA purity was assessed on 1% agarose gels. Extracted DNA was stored at −20°C for later use. Samples with a bright main band at 400–450 bp were chosen for further analysis. Next, the DNA was diluted to 1 ng/μl using sterile water. Thereafter, sequencing libraries were generated using a TruSeq^®^ DNA PCR-Free Sample Preparation Kit (Illumina, Inc., San Diego, CA, USA) following the manufacturer's recommendations, and index codes were added. Briefly, 250 ng of DNA was end repaired, purified with AmpureXP beads (Agencourt; Beckman Coulter), adenylated, and adapters ligated. The libraries were cleaned up and size-selected to remove adapter monomers and dimers. The library quality was assessed using a Qubit^®^ 2.0 Fluorometer (Life Technologies, ThermoFisher Scientific, USA) and a 2100 Bioanalyzer system (Agilent Technologies, Inc., Santa Clara, CA, USA). Lastly, the library was sequenced on a HiSeq X ten platform (Illumina), generating 150-bp paired-end reads.

### Metagenome Assembly and Non-redundant Gene Set Prediction and Annotation

According to the unique barcode and primer sequences, paired-end reads were accurately assigned to the relevant samples. High-quality clean reads were generated by removing the adaptor sequences and low-quality sequences (default low quality threshold: 5, default low quality rate: 0.5, and default N rate threshold: 0.05), and PCR duplications were removed. Host DNA was removed according to the Human Sequence Removal protocol of the National Center for Biotechnology Information (NCBI) using a pig reference genome (Sscrofa11.1). The clean reads were assembled into contigs using megahit (v1.0.6) (18). Open reading frames (ORFs) were predicted by prodigal (v2.6.3) and the clean reads were aligned to ORFs to calculate the mapped read count. Thereafter, the ORFs were clustered to remove redundancy for building a set of representative genes through CD-HIT software (v4.7). To avoid bias related to variation in the ORF size, both ORF sequence length and sequencing depth were included in the data normalization process prior to statistical comparisons. The normalized abundance matrix (G) was calculated for all samples:

$$\begin{array}{l} G={\left(g_{kh}\right)}_{m\times n}=\left[\begin{array}{ccc} g_{11} & \cdots & g_{1m}\\ \vdots & \ddots & \vdots \\ g_{n1} & \cdots & g_{nm} \end{array}\right],\;g_{kh}=\frac{\sum_{i=1}^{p}\frac{D_{ki}}{L_{ki}}}{\sum_{h=1}^{n}\sum_{i=1}^{p}\frac{D_{ki}}{L_{ki}}} \end{array}$$

where m is the number of samples, *n* is the number of representative genes, g_kh_ is the normalized abundance of representative gene h for sample k, p is the total number of ORFs clustered for representative gene h and sample k, D_ki_ is the mapped reads count of ORF i for sample k, and L_ki_ is the length of ORF i for sample k.

ORFs were annotated with taxonomic information using Diamond based on the NCBI-nr database (cutoff *E*-value ≤ 10^−6^). The annotated results were revised by MEGAN6 using the lowest common ancestor algorithm. Further, we specially identified the ORFs related to pathogenic bacteria based on the common pathogenic bacteria list of our laboratory (Supplementary Table S1) according to the above ORFs taxonomic information. Subsequent diversity analyses were all performed based on normalized taxonomy abundance. ORFs were annotated with functional information using the Kyoto Encyclopedia of Genes and Genomes (KEGG) Automatic Annotation Server (KAAS) (19) based on the KEGG database with default parameters.

### ARG and MGE Analysis

ARGs were characterized by mapping ORFs to the Comprehensive Antibiotic Resistance Database (CARD) (V3.0.0) using the Resistance Gene Identifier (RGI) application. The CARD is a rigorously curated collection of characterized, peer-reviewed antibiotic resistance determinants (20). We identified the 16S rRNA genes in the ORF gene set by aligning the ORFs to the SILVA database (v132). The ARG count data were normalized based on the 16S rRNA gene copy number in order to express the ARG abundance as “ARG copy per 16S rRNA gene copy”, as suggested by Li et al. using the following formula:

$$\begin{array}{l} A={\left(a_{uv}\right)}_{x\times y}=\left[\begin{array}{ccc} a_{11} & \cdots & a_{1x}\\ \vdots & \ddots & \vdots \\ a_{y1} & \cdots & a_{yx} \end{array}\right],\;a_{uv}=\frac{\sum_{j=1}^{q}G_{uj}}{\sum G_{16S}} \end{array}$$

where x is the number of samples, y is the number of ARGs, a_uv_ is the normalized abundance of ARG v for sample u, q is the total number of representative genes annotated to ARG v of sample u, G_uj_ is the normalized abundance of representative gene j for sample u, and $\sum_{j=1}^{q}G_{uj}$ means the abundance sum of all representative genes annotated to ARG v of sample u. ∑G_16s_ is the total normalized abundance of the 16S rRNA gene for sample u.

Transferrable ARGs (T-ARGs) are typically of greater clinical concern than non-transferable ARGs. To specifically assess the T-ARGs, a separate ARG database, ResFinder (v3.2), which focuses on acquired ARGs, was used. Briefly, ORFs were submitted to the ResFinder webpage (https://cge.cbs.dtu.dk/services/ResFinder/) and the identity threshold was set to ≥90% with ≥60% minimum length match. T-ARG count data were normalized based on the 16S rRNA gene copy number (as for the ARG count data).

Diamond was used to map the ORFs to the MGE database. The MGE database contains genes with 278 different gene name annotations (annotated as IS^*^, ISCR^*^, intI1, int2, istA^*^, istB^*^, qacEdelta, tniA^*^, tniB^*^, tnpA^*^, and Tn916 transposon in the NCBI nucleotide database) and more than 2,000 unique sequences, excluding the sequences from the PlasmidFinder database (12). Subsets of ORFs that were mapped to MGEs were used to produce the MGE profiles. MGE count data were also normalized based on the 16S rRNA gene copy number (as for the ARG count data).

In order to identify the ARGs and MGEs in pathogenic bacteria, the ORFs related to pathogenic bacteria were chose and were calculated according to the above methods.

### Statistical Analysis

Alpha diversity analysis, which indicates the complexity of species diversity for each sample, was conducted using R package VEGAN (V2.5-3). The alpha diversity of different groups was compared using Wilcoxon rank-sum tests. Beta diversity was calculated based on the Bray–Curtis distance and weighted UniFrac distance using the R package VEGAN (V2.5-3) (21). Differences in beta diversity were identified using analysis of similarity (ANOSIM), with the effect size being indicated by an *R*-value [between −1 and +1, with 0 indicating that the null hypothesis cannot be rejected (22)], and permutational multivariate analysis of variance (PERMANOVA) leveraged by stress, with the effect size being indicated by R^2^ (between 0 and 1). Differences in community structure based on beta diversity were visualized using principal coordinate analysis (PCoA) with the R package ape and the non-metric multi-dimensional scaling (NMDS) method using the R package VEGAN. Significantly different phylum and genus were identified between LAD and CON groups for colon and ileum using STAMP (v2.1.3) (23), respectively. The correlation analysis between pathogeny microbes and cytokines was performed using R package corrplot. Statistical significance was defined as *P* < 0.05 for all analyses.

## Results

### Effects of LDA on Systemic and Intestinal Immunity

**Figure 1:**
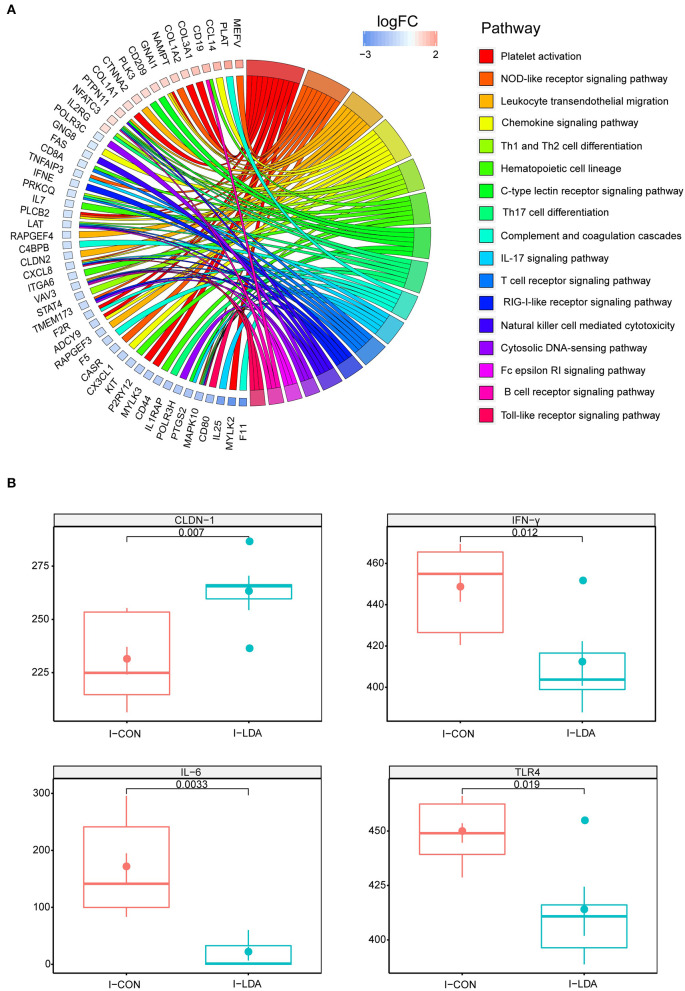
Intestinal immunity analysis between I-LDA, and I-CON groups. **(A)** GOChord plot displays the relationship between a list of pathway and corresponding genes. The DEGs located in plot left were ordered according to their logFC. The GO terms located in plot right were cluster as server category. **(B)** Between-group comparisons of four immune-related proteins using two-sided independent Wilcoxon tests. I-CON, ileum subgroup of CON group; I-LDA, ileum subgroup of LDA group.

To investigate the effects of LDA on immunity in weaned piglets, we first assessed the serum immunological parameters. No significant differences in the levels of IgG, IgM, complement C3, and C- Reactive Protein (C-RP) were observed in the serum samples between the LDA and CON groups (Supplementary Figure S1). Considering that LDA exposure induced significantly reduced total bacterial load in the ileum content, we then focused on the transcriptomics analyses of the ileum tissues by RNA-seq technology. A total of 22.98 ± 1.96 million clean reads for each sample were generated from ileum tissues of 19 piglets, of which 89.67 ± 1.72% could be aligned to the pig reference genome (Supplementary Table S2). Based on these data, pair-wise comparisons between the LDA group and CON groups revealed 52 immune-related differentially expressed genes (DEGs) out of totally 1,247 DEGs (FC > 1.5, *p* < 0.05) (Supplementary Table S3). KEGG enrichment analysis indicated that these immune-related DEGs were enriched in 17 pathways, majority of which were innate-immunity pathways (Figure 1A; Supplementary Table S4). Thereby, we further analyzed the protein expression of 10 innate immunity-related genes, including four cytokines, by ELISA in ileum tissues of piglets (Figure 1B; Supplementary Figure S2). The results showed that the LDA group had significantly decreased levels of *IFN-*γ*, IL-6*, and *TLR4* (all *P* < 0.05), suggesting that LDA exposure for 4 weeks may induce an early anti-inflammatory response. In addition, the *CLDN-1* protein expression was significantly elevated (*P* < 0.05), indicated that the intestinal barrier might be improved upon LDA exposure for 4 weeks.

### Community Structure of ARG-Containing Bacteria

Deep metagenomic sequencing was then performed on 23 samples of ileum content (10 LDA samples and 13 CON samples) and 24 samples of colon content (11 LDA samples and 13 CON samples) of the weaned piglets (Supplementary Table S5). From these samples, 456 Gb of high-quality reads, with an average of 9.7 Gb per sample, were generated. The clean data from each sample allowed successful assembly, with a mean N50 (minimum contig length needed to cover 50% of the genome) of 2.09 kb. Thereafter, a total of 5,306,978 ORFs were obtained. We obtained a similar bacterial community structure as in our previous study (which was based on 16S rRNA sequencing), which indicated apparent differences (based on the Shannon index) between the colon and ileum samples, while no clear structural divergence was found between the LDA and CON groups (Supplementary Figure S3). A total of 939,519 core ORFs were obtained, comprising 48,668 in the colon subgroup of the LDA group (C-LDA), 63,749 in the colon subgroup of the CON group (C-CON), 1,707 in the ileum subgroup of the LDA group (I-LDA), and 10,569 in the ileum subgroup of the CON group (I-CON).

**Figure 2:**
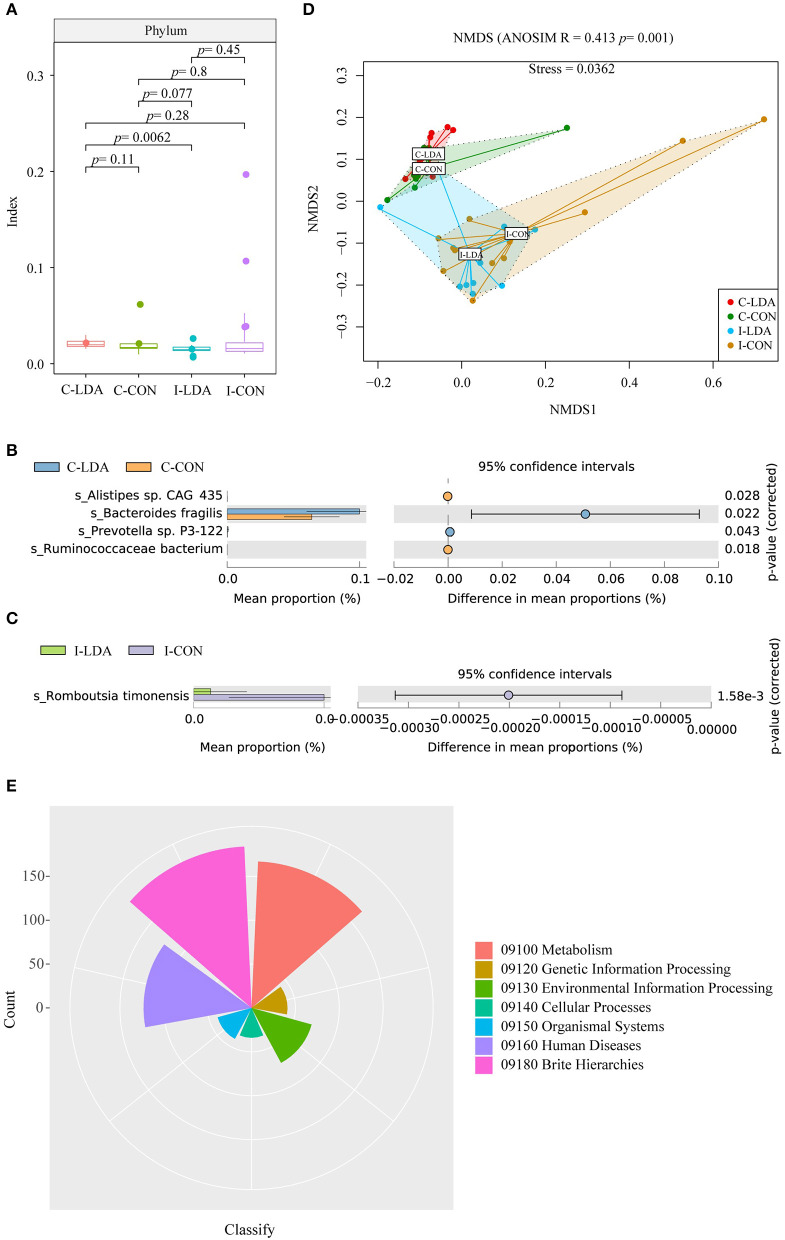
Analysis of the community structure of the resistome-containing bacteria among the C-LDA, C-CON, I-LDA, and I-CON groups. **(A)** Alpha diversity analysis of the resistome-containing bacteria in all four groups using the Shannon index. Species with significantly different (*P* < 0.05) mean abundances (using two-sided Welch's *t*-tests) between the **(B)** C-LDA and C-CON groups and **(C)** I-LDA and I-CON groups, based on SILVA database annotation. **(D)** Beta diversity analysis of the functions of four groups using NMDS analysis based on Bray–Curtis distance among all samples. Analysis of similarity (ANOSIM) was conducted, with the effect size being indicated by an R-value (between −1 and +1, with 0 indicating that the null hypothesis cannot be rejected). **(E)** KEGG pathway annotation in all four groups. C-CON, colon subgroup of control (CON) group; C-LDA, colon subgroup of low-dose antibiotic (LDA) group; I-CON, ileum subgroup of CON group; I-LDA, ileum subgroup of LDA group.

Based on this gene set, we further identified 4941 ORFs that comprised the gut resistomes, involving ARGs and MGEs. The bacteria that contained these ORFs were then identified. There were no clear differences in alpha diversity between the C-LDA and C-CON groups or the I-LDA and I-CON groups (Figure 2A). Beta diversity analysis indicated for clear divergence between colon and ileum samples (ANOSIM *R* = 0.413, *P* = 0.001), but not between the LDA and CON groups of colon or ileum.

*Escherichia coli* was the most abundant resistome-containing species in the ileum samples, while *Bacteroides fragilis* was the most abundant resistome-containing species in the colon samples. *Alistipes* sp. CAG_435 and *Ruminococcaceae bacterium* were significantly decreased and *Bacteroides fragilis* and *Prevotella* sp. P3-122 were significantly increased in the C-LDA group compared to the C-CON group (all *P* < 0.05). *Romboutsia timonensis* was significantly decreased (*P* = 1.58e^−3^) in the I-LDA group compared to the I-CON group (Figures 2B,C). Furthermore, KEGG pathway annotations were assigned to the four groups (Figures 2D,E).

### Changes in Antibiotic Resistance After LDA Exposure

**Figure 3:**
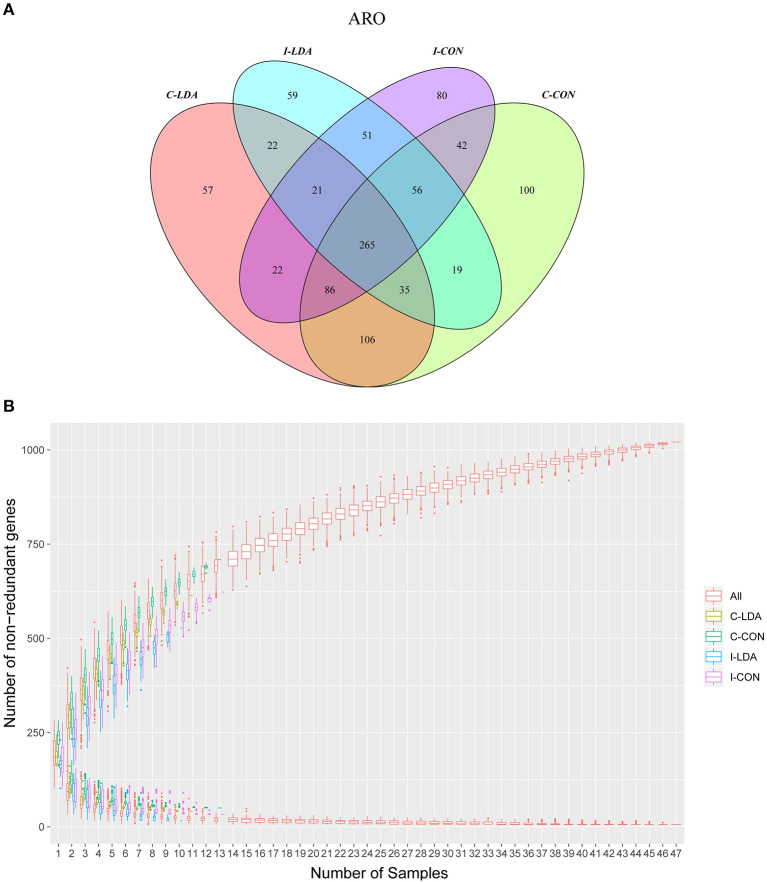
Antimicrobial resistance gene (ARG) catalog analysis between the C-LDA, C-CON, I-LDA, and I-CON groups. **(A)** Venn diagram showing distribution of the ARGs in the four groups. **(B)** Rarefaction analysis of pan and core resistome genes including all samples in each group. C-CON, colon subgroup of control (CON) group; C-LDA, colon subgroup of low-dose antibiotic (LDA) group; I-CON, ileum subgroup of CON group; I-LDA, ileum subgroup of LDA group.

In total, 1,021 ARGs were detected across all samples, which belonged to 182 gene families (Supplementary Tables S6, S7; Supplementary Figures S4A,B). Only 265 (26%) ARGs were common among the four groups. Between the I-CON and I-LDA groups, 393 ARGs were common and between the C-LDA and C-CON groups, 492 ARGs were common (Figure 3A). There were no significant differences in the total amount, structure, or diversity of ARGs between the I-LDA and I-CON groups or the C-LDA and C-CON groups. Furthermore, the highly individual-specific composition of ARGs was revealed by a rarefaction analysis of the pan and core resistome genes (Figure 3B).

**Figure 4:**
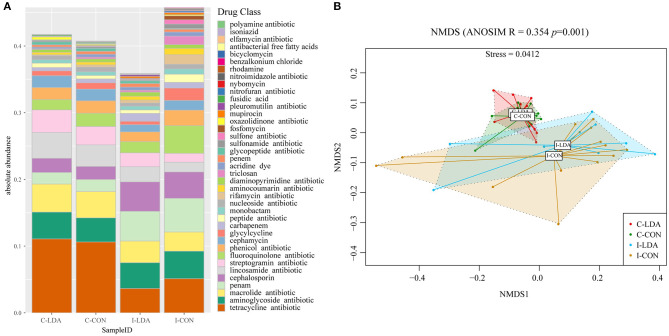
Composition and structure of antimicrobial resistance genes (ARGs) between the C-LDA, C-CON, I-LDA, and I-CON groups. **(A)** Absolute abundance of antibiotic resistance classes in the four groups. **(B)** Beta diversity analysis of antibiotic resistance classes in the four groups using NMDS analysis based on Bray–Curtis distance. Analysis of similarity (ANOSIM) was conducted, with the effect size being indicated by an *R*-value (between −1 and +1, with 0 indicating that the null hypothesis cannot be rejected). C-CON, colon subgroup of control (CON) group; C-LDA, colon subgroup of low-dose antibiotic (LDA) group; I-CON, ileum subgroup of CON group; I-LDA, ileum subgroup of LDA group.

Over 50.66% of the ARGs in the samples were predicted to confer multidrug resistance (involving up to six antibiotic resistance mechanisms). Overall, the ARGs were predicted to confer resistance to 39 classes of antibiotics, with a range of 0–0.14 ARG copies per 16S rRNA gene copy among the samples. The top five most abundant antibiotic resistance classes were tetracycline, macrolide, aminoglycoside, lincosamide, and streptogramin in the C-LDA and C-CON groups, and tetracycline, penam, fluoroquinolone, aminoglycoside, and cephalosporin in the I-LDA and I-CON groups (Figure 4A). NMDS analyses indicated that the antibiotic resistance classes were significantly different between the C-LDA and I-LDA groups and the C-CON and I-CON groups (ANOSIM *R* = 0.354, *P* = 0.001) (Figure 4B). The differences of ARG levels between colon and ileum samples (i.e., between the C-LDA and I-LDA groups and the C-CON and I-CON groups) were larger than between the LDA and CON groups (i.e., between the I-LDA and I-CON groups and the C-LDA and C-CON groups) (Supplementary Figure S4C).

Pair-wise comparisons revealed only one and two significantly different antibiotic classes (based on the abundance of ARGs) between the C-LDA and C-CON groups and the I-LDA and I-CON groups, respectively, and there were no significantly different antibiotic mechanisms. Rifamycin resistance was the only significantly different antibiotic resistance class (*P* < 0.05) between the C-LDA and C-CON groups; it involved 30 ARGs, which had non-significant differences between the C-LDA and C-CON groups. Oxazolidinone and sulfone resistance were the only significantly different antibiotic resistance classes (all *P* < 0.05) between the I-LDA and I-CON groups (Supplementary Table S8). Eight and three ARGs were predicted to confer the oxazolidinone and sulfone resistances, respectively, and three of the oxazolidinone resistance genes were significantly different (all *P* < 0.05) between the I-LDA and I-CON groups (Supplementary Figure S5). We also revealed a number of differential ARG gene families and ARGs (Supplementary Tables S9, S10).

### Dissemination of Antibiotic Resistance After LDA Exposure

**Figure 5:**
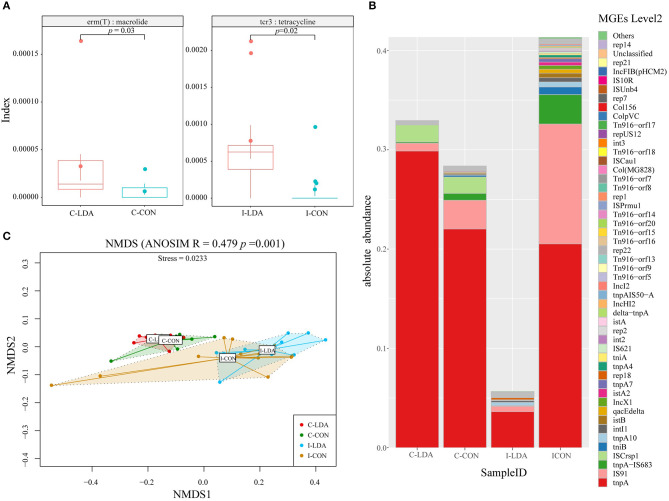
Comparison of antibiotic resistance elements between the C-LDA, C-CON, I-LDA, and I-CON groups. **(A)** Between-group comparisons of two T-ARGs using two-sided independent Wilcoxon tests. **(B)** Absolute abundance of mobile gene elements (MGEs; level 2) in the four groups. **(C)** Beta diversity analysis of MGEs in the four groups using NMDS analysis based on Bray–Curtis distance. Analysis of similarity (ANOSIM) was conducted, with the effect size being indicated by an *R*-value (between −1 and +1, with 0 indicating that the null hypothesis cannot be rejected). C-CON, colon subgroup of control (CON) group; C-LDA, colon subgroup of low-dose antibiotic (LDA) group; I-CON, ileum subgroup of CON group; I-LDA, ileum subgroup of LDA group.

We examined two elements that greatly contribute to the dissemination of antibiotic resistance: T-ARGs and MGEs. The representative ORFs were aligned to a ResFinder database containing only acquired ARGs and a customized MGE database. As a result, 148 T-ARGs were detected, conferring resistance to 12 classes of antibiotics, mainly including aminoglycoside, beta-lactam, tetracycline, macrolide, and phenicol. Among them, there was one significantly different T-ARG between the C-LDA and C-CON groups and four significantly different T-ARGs between the I-LDA and I-CON groups. Erm(T) was significantly increased (*P* < 0.05) in the C-LDA group compared to the C-CON group, whereas, cfr(B), aac (3)-IId, cfxA6, and tcr3 had significant differences (*P* < 0.05) between the I-LDA and I-CON groups (Figure 5A). Notably, the T-ARGs Erm(T) and tcr3 confer macrolide and tetracycline resistance, respectively, which reflected the LDA drugs used in this study (i.e., the virginiamycin contains macrolide and the chlortetracycline belongs to tetracycline). This confirms that LDA use significantly enriches the T-ARGs related to the specific antibiotic classes administered.

MGEs can transfer ARGs between members of the gut microbial community (24). In total, 129 MGEs (level 2) were detected across all four groups, while 38 (29%) MGEs were common to the four groups. Between the I-LDA and I-CON groups, 83 MGEs were common, and between the C-LDA and C-CON groups, 47 MGEs were common (Supplementary Figure S6). Among these MGEs, tnpA was the most abundant MGE in all four groups, followed by IS91, tnpA-IS683, and ISCrsp1 in C-LDA and C-CON groups; IS91, tnpA-IS683, and tnpA10 in I-CON group; IS91, tnpA10, and rep18 in I-LDA group (Figure 5B). NMDS analyses indicated that the MGEs (level 2) were not significantly different between the C-LDA and C-CON groups (ANOSIM *R* = 0.0592, *P* = 0.106) or the I-LDA and I-CON groups (ANOSIM *R* = −0.0214, *P* = 0.508) (Supplementary Figure S7). In contrast, MGE differences between colon and ileum samples within group (i.e., between the C-LDA and I-LDA groups and the C-CON and I-CON groups) were larger than between the LDA and CON groups (i.e., between the I-LDA and I-CON groups and the C-LDA and C-CON groups) (Figure 5C). Pair-wise comparisons revealed that the abundance of nine MGEs (level 2) were significantly decreased (all *P* < 0.05) in the C-LDA group compared to the C-CON group, comprising int3, rep18, Tn*916–orf14*, Tn*916–orf15*, Tn*916–orf16*, Tn*916–orf17*, Tn*916–orf18*, Tn*916–orf20*, and Tn*916–orf7* (Supplementary Table S11). Additionally, five significantly different MGEs (level 2) were identified (all *P* < 0.05) in the ileum analysis, comprising three MGEs that were significantly decreased (int3, Tn*916–orf7*, and rep14) and two MGEs that were significantly increased (tnpA1B1–IS1068, and tnpA3B3–ISLL6) in the I-LDA group compared to the I-CON group (Supplementary Table S12).

### Antibiotic Resistomes–Pathogenic Bacteria–Innate Immunity Interactions

To examine the antibiotic resistance status of specific pathogenic bacteria, we matched the identified ARGs to common enteral pathogens and identified four pathogens in the samples (Supplementary Figure S8), comprising *Campylobacter coli, Streptococcus suis, Clostridium perfringens*, and *Klebsiella pneumoniae*. Thereafter, we found that *Campylobacter coli*, which is a commensal in the gastrointestinal tract but can sometimes cause enteritis in pigs and humans, was significantly increased in the I-LDA group (*P* = 0.014) compared to the I-CON group. Additionally, *Streptococcus suis*, which is a zoonotic pathogen that is an emerging threat to human health (25), was significantly decreased in the C-LDA group (*P* = 0.0099) compared to the C-CON group.

*Campylobacter coli*, which was the significantly different pathogenic bacterial species between the I-LDA and I-CON groups, was only matched to one ARG [APH(2”)-If]. There were no significant differences in antibiotic resistance (based on the abundance of ARG) in *Campylobacter coli* between the I-LDA and I-CON groups. The ARG in *Campylobacter coli* was predicted to confer aminoglycoside resistance. There were no differences in MGEs in *Campylobacter coli* between the I-LDA and I-CON groups.

*Streptococcus suis*, which was the significantly different pathogenic bacterial species between the C-LDA and C-CON groups, was matched to two ARGs, comprising SAT-4, and Erm(T). There was no significant difference in these ARGs between the C-LDA and C-CON groups. The ARGs in *Streptococcus suis* were predicted to confer macrolide, lincosamide, nucleoside, and streptogramin resistance and one ARG [Erm(T)] had transfer potential. However, pair-wise comparisons revealed no significant differences in antibiotic resistance (based on the abundance of ARGs) in *Streptococcus suis* between the C-LDA and C-CON groups. Five MGEs were detected in *Streptococcus suis*, comprising tnpA, repUS17, rep18, rep22, and Tn*916-orf7*. Among them, there was one significantly different MGE, Tn*916-orf7*, in *Streptococcus suis* between the C-LDA and C-CON groups.

**Figure 6:**
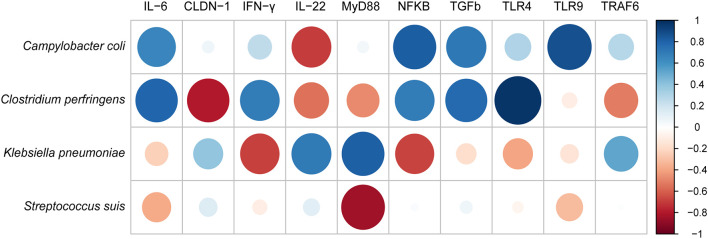
The correlation analysis between pathogenic bacteria and innate immune cytokines measured by ELISA using corrplot package of R.

We further performed the correlations between identified four pathogens in the samples and the 10 immune-related proteins (Figure 6). As a result, *Campylobacter coli* has strongly positive correlation with *IL-6, NF-*κ*B, TGF-*β and *TLR9*, and negative correlation only with *IL-22*. Whereas, both *Clostridium perfringens* and *Klebsiella pneumoniae* were positively correlated with almost half of cytokines. In contrast, *Streptococcus suis* was only negatively correlated with *MyD88, IL-6*, and *TLR9*.

## Discussion

High-dose antibiotics are used for the clinical management of diseases in humans and animals, and LDAs are used as feed additives to promote the growth of livestock. The use of high-dose antibiotics enriches ARGs, which may lead to an uncoupling of the mutualistic relationships that have evolved over long periods between the gut microbiota and the host (26, 27). LDA accumulation is believed to disrupt the microbiota composition and consequently promote host growth (28–30). Furthermore, LDA residue in breastmilk can be transferred to the infant gut (6), and therefore LDA may also affect the gut microbiota of infants. Using a pig model of LDA exposure for 4 weeks, our study aimed to evaluate the effects of LDA on intestinal immunity and antibiotic resistance, including ARGs and MGEs, based on colon and ileum samples, over a relatively short period. In general, we revealed the intestinal innate immunity was considerably affected upon LDA exposure, as well as the highly individual-specific diversity regarding the resistomes and, probably because of this, the structure and diversity of the resistomes were not significantly different between the I-LDA and I-CON groups or C-LDA and C-CON groups.

Despite the highly individual-specific diversity, we identified significantly different antibiotic classes (based on the abundance of ARGs and MGEs) between the LDA and CON groups. Notably, although the ARG divergence was large, the classes and mechanisms of antibiotic resistance were highly consistent between the ileum and the colon. This suggested that the microbiota and ARGs, but not the overall resistomes, were different between different intestinal segments. Furthermore, T-ARGs that were predicted to confer macrolide and tetracycline resistance, including Erm(T) and tcr3, respectively, were revealed to be significantly increased after LDA exposure in the colon and ileum samples. Therefore, even over a relatively short period of 4 weeks, levels of antibiotic resistance to administered antibiotics can be changed. Two previous studies reported negligible impacts of LDA on antibiotic resistance based on testing fecal microbiota over both long and short periods (31, 32). There were between-study differences in the antibiotics, source of microbiota, and time period, and our results suggest that a more comprehensive evaluation should be conducted before concluding that the impacts of LDA are negligible.

We not only identified antibiotic resistance in the LDA group, but also in the CON group not exposed to antibiotics, and both groups exhibited highly individual-specific diversity. The infant's gut microbiota is shaped by many factors (33–35), but the most studied aspect is the correlation of the gut microbiota between infants and mothers (36, 37). It has been reported that infants derive their first microbiota from their mother and so it is very likely that infants share gut resistomes (including MGEs) with their mother's gut and breastmilk microbiota during pregnancy, birth, and lactation (38, 39), which may be acquired from the mother. Additionally, the resistomes of the infants might be influenced by the antibiotics in the breastmilk, which may also underlie the highly individual-specific diversity of the gut resistomes in infants.

The composition of the microbiota affects immune responses, susceptibility to infection by intestinal pathogens, and the development of inflammatory bowel diseases (40), while antibiotics alter the intestinal microbiota and thereby influence the intestinal immune defenses, which can involve reductions or increases in the expression of intestinal innate immunity-related genes (41, 42). In this study, there were two bacterial pathogens with significantly different abundances between the I-LDA and I-CON groups and C-LDA and C-CON groups (based on matching the ARGs to common enteral bacterial pathogens), which composed limited ARGs in the pathogens with non-significant differences in abundances. Further, the intestinal immunity was considerably changed between the LDA and CON groups. This revealed that with short-term LDA exposure, the changes in intestinal immunity were considerable, probably because of the changes in pathogenic bacterial species and the early anti-inflammation response [significantly decreased *TLR4* and *two* pro-inflammatory cytokines (*IFN-*γ and *IL-6*)] and improved intestine barrier (significantly increased *CLDN-1*). Moreover, the correlations between bacterial pathogens and immune-related proteins indicated differential abundance of specific gut bacteria was associated with cytokine responses, it has been reported that this effect was exerted directly on the intrinsic cytokine production capacity (43).

Overall, our findings revealed considerable changes in intestinal immunity, but minimal changes in the gut antibiotic resistomes (ARGs and MGEs), in weaned piglets after LDA exposure for 4 weeks. Our study focused on the short-term impact of LDA on the colon and ileum resistomes and intestinal immunity in the first 4 weeks after weaning. As the duration of LDA exposure in this study was only 4 weeks, the antibiotic resistome profiles reported here may not be representative of the longer-term effects. It would be interesting to continue to assess piglets for a longer period after weaning to investigate how the minor changes impact the future health and productivity of growing piglets.

## Data Availability Statement

The datasets generated in this study are available in the NCBI Sequence Read Archive (SRA) repository. The metagenome data is linked to accession numbers SRR11489746-SRR11489792. The strand-specific RNA-seq data is linked to accession numbers SRR7779786-SRR7779804.

## Ethics Statement

The animal study was reviewed and approved by the Sichuan Agricultural University animal welfare committee.

## Author Contributions

FG and LC conceived of and designed the study. RW, LQ, and QX collected samples and performed the animal experiments. RW performed the qPCR experiment. QH and DZ processed and analyzed the data. FG, QH, and CL interpreted the data and wrote the manuscript. All authors read and approved the final manuscript.

## Conflict of Interest

The authors declare that the research was conducted in the absence of any commercial or financial relationships that could be construed as a potential conflict of interest.
