# Supplementary material for: Effects of Low-Dose Antibiotics on Gut Immunity and Antibiotic Resistomes in Weaned Piglets
Source: Front Immunol. 2020 Jun 10;11:903. doi: 10.3389/fimmu.2020.00903 (PMC7325945; doi:10.3389/fimmu.2020.00903)
Supplement: Supplementary file 1 [file Data_Sheet_1.PDF]

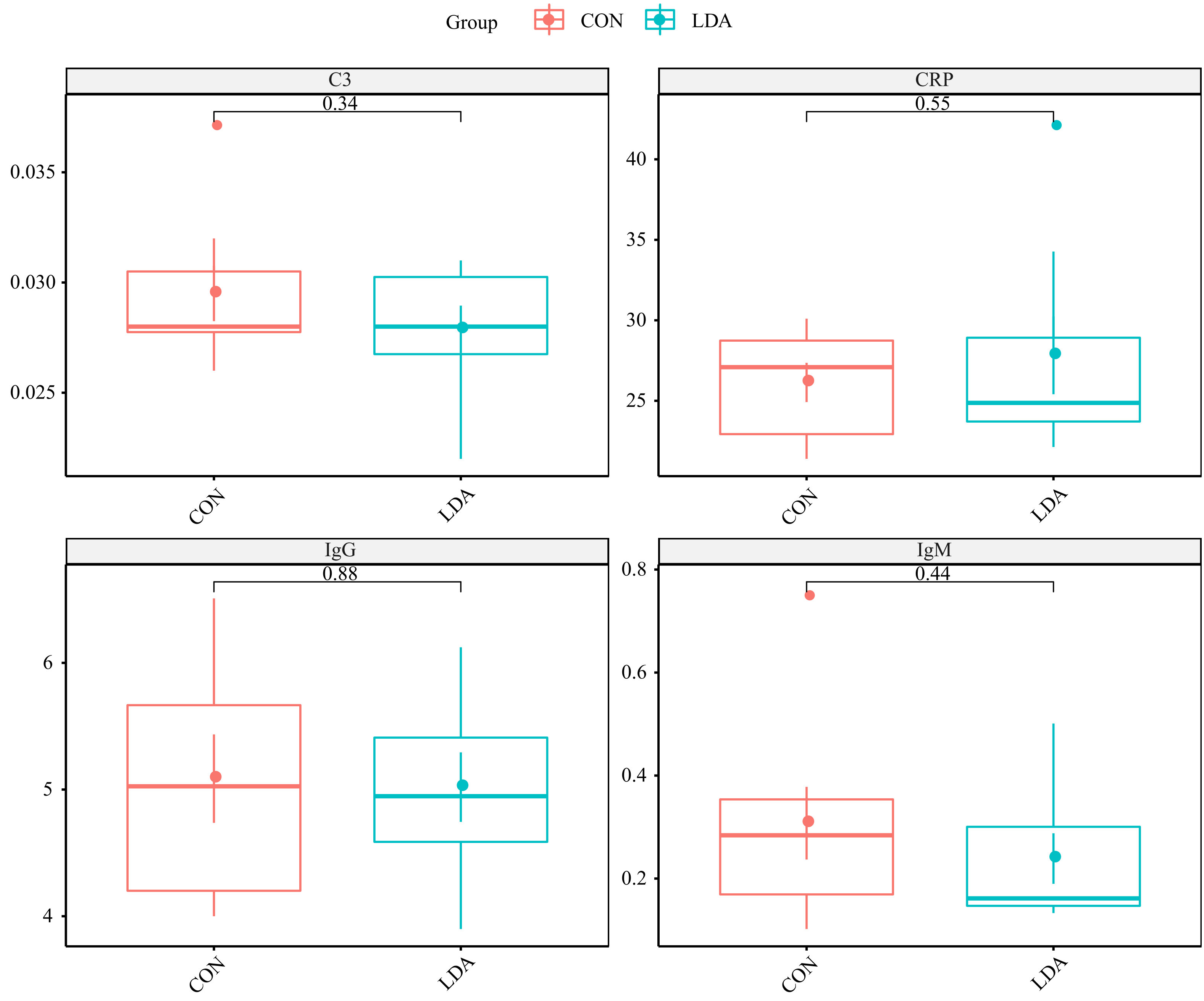

Supplementary Figure S1. The concentrations (mg/L) of C3, CRP, IgG, IgM in serum with or without LDAexposure. CON: content of control group; LDA: content of low-dose antibiotics treatment group.

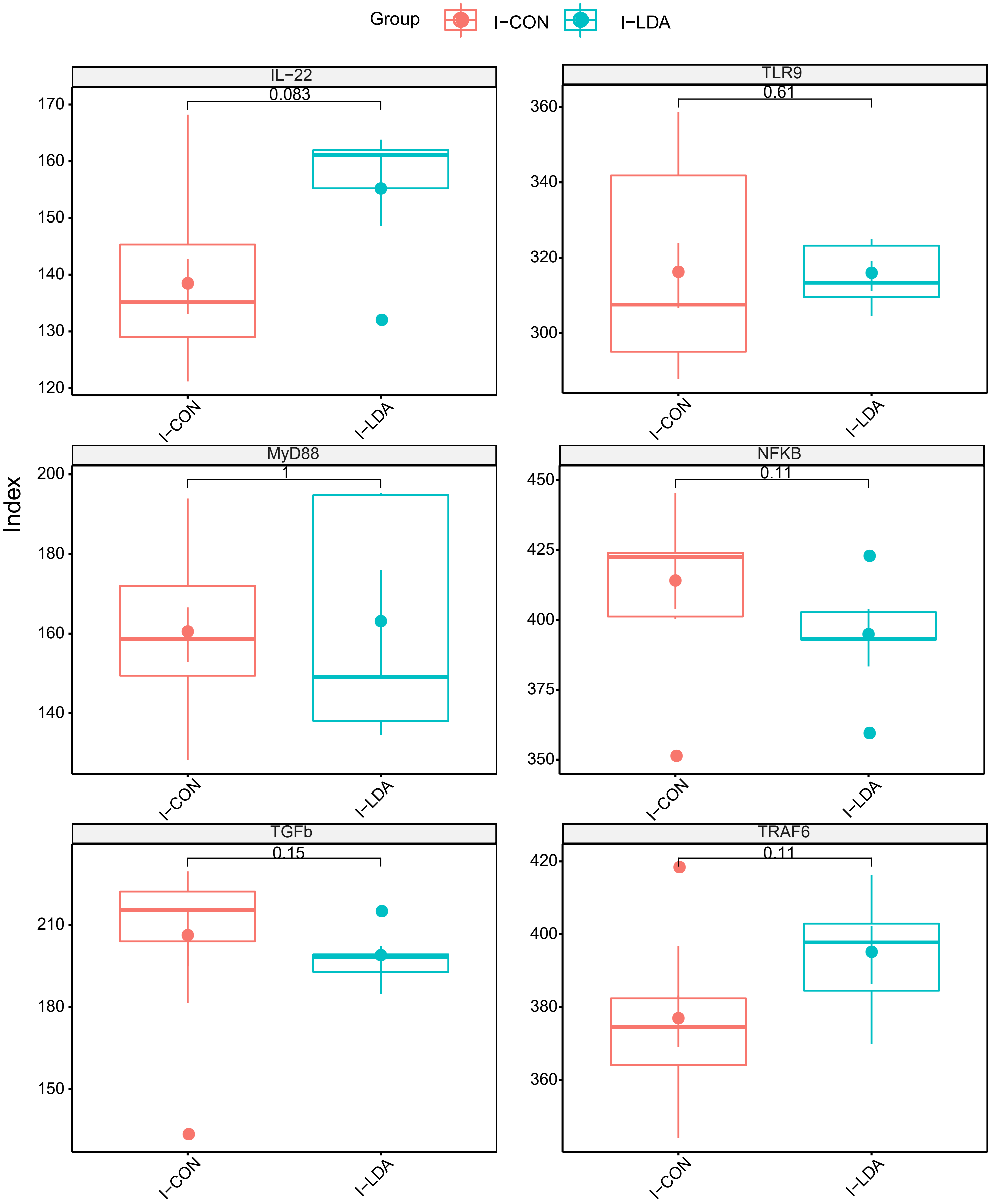

Supplementary Figure S2. The difference analysis of intestinal innate immune cytokines levels with or without LDAexposure. CON: content of control group; LDA: content of low-dose antibiotics treatment group.

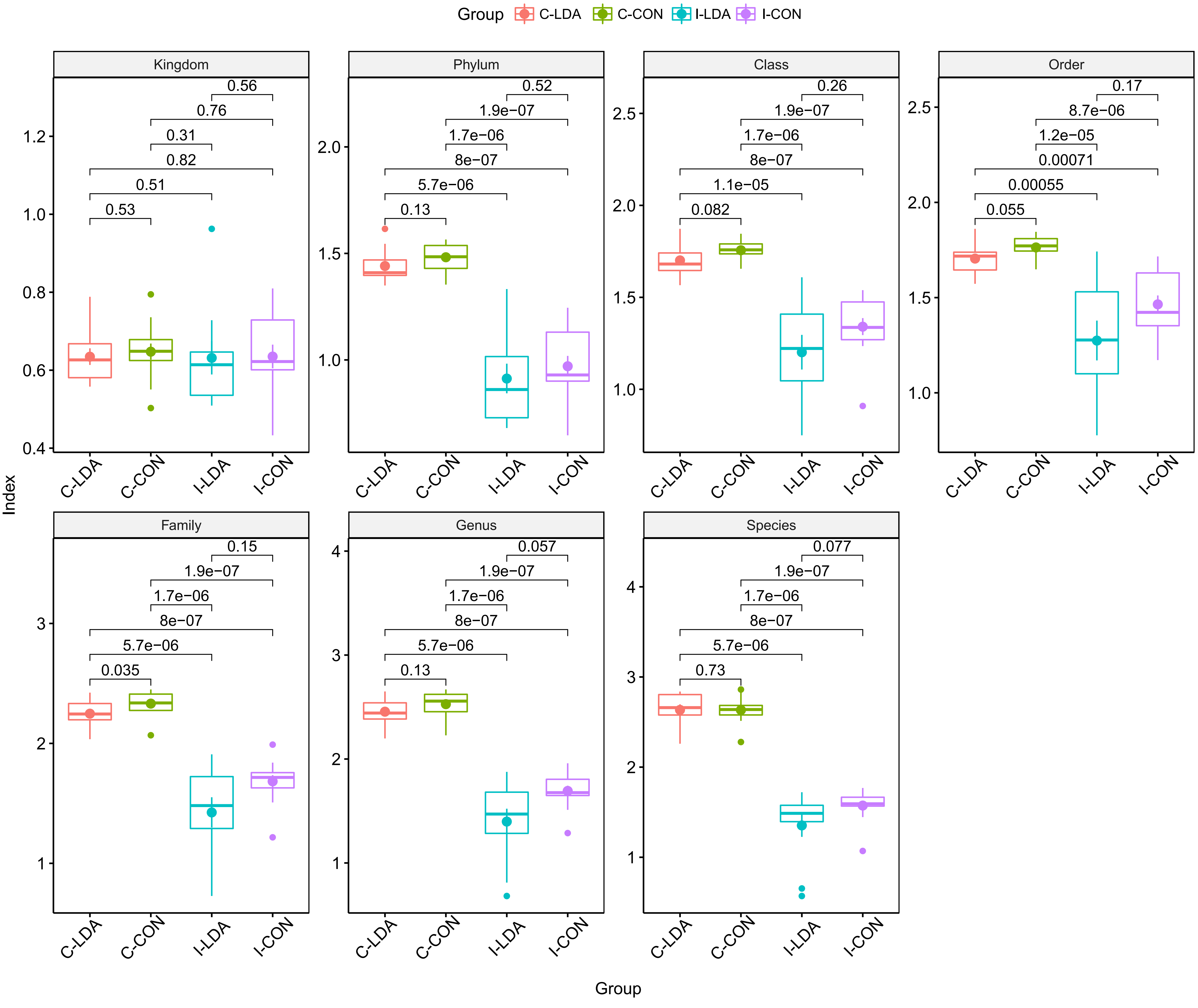

Supplementary Figure S3. Alpha analysis of microbiota in all groups using Shannon index.

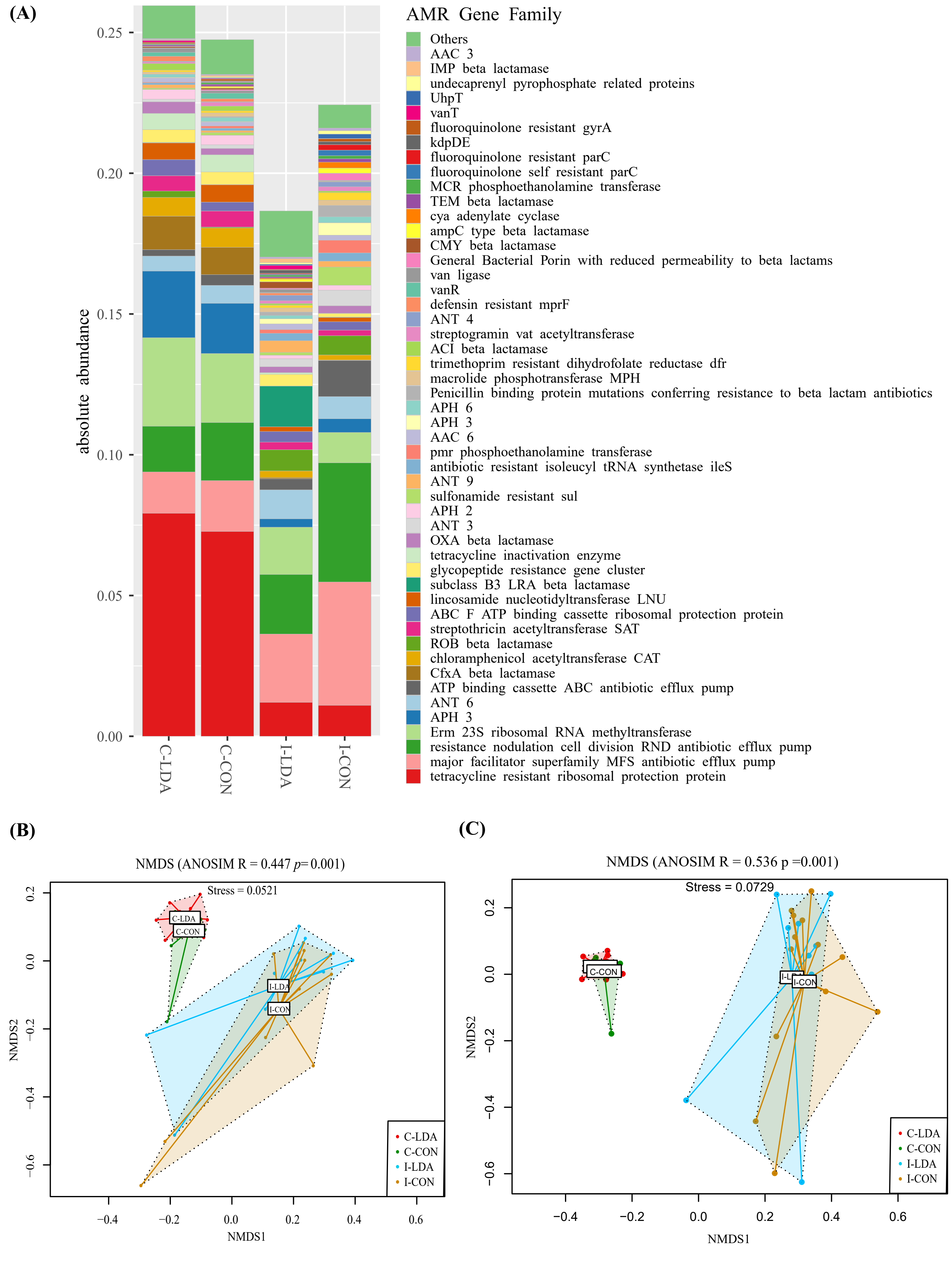

Supplementary Figure S4. The composition and structure of ARGs family in the colon and ileum contents betweenLDA and CON groups. (A) the absolute abundance of ARGs family in four groups. (B) Beta diversity analysis of ARGsfamily in four groups using NMDS analysis based on Bray-Curtis distance of Features. Analysis of Similarity (ANOSIM)and effect size was indicated by an R-value (between −1 and +l, with a value of 0 representing the null hypothesis). (C) Beta diversity analysis of ARGs in four groups using NMDS analysis based on Bray-Curtis distance of Features.Analysis of Similarity (ANOSIM) fect size was indicated by an R-value (between −1 and +l, with a value of 0 representingthe null hypothesis).

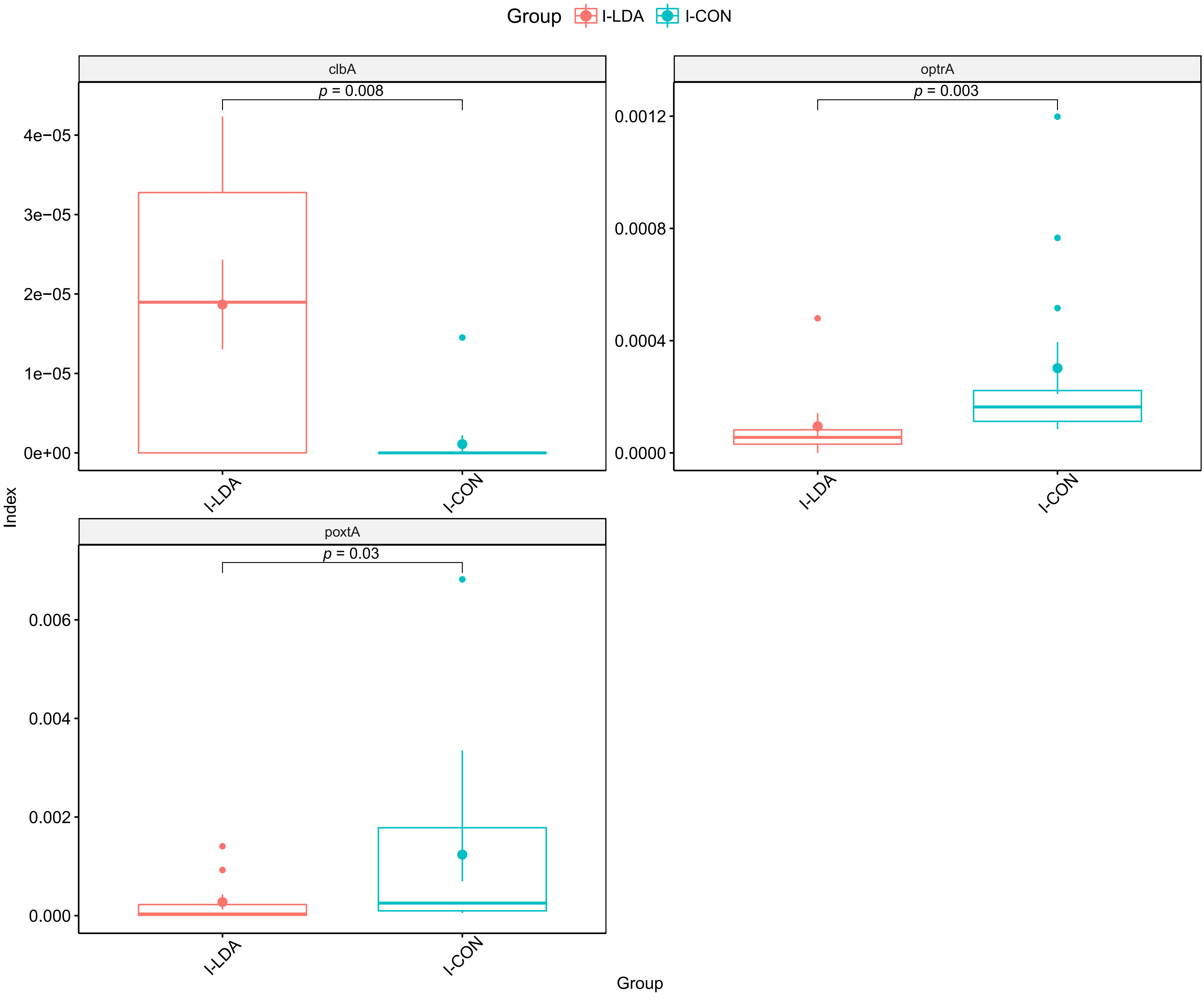

Supplementary Figure S5. The differential analysis of ARGs conferred the oxazolidinone resistance using two-sided independent wilcox-test method.

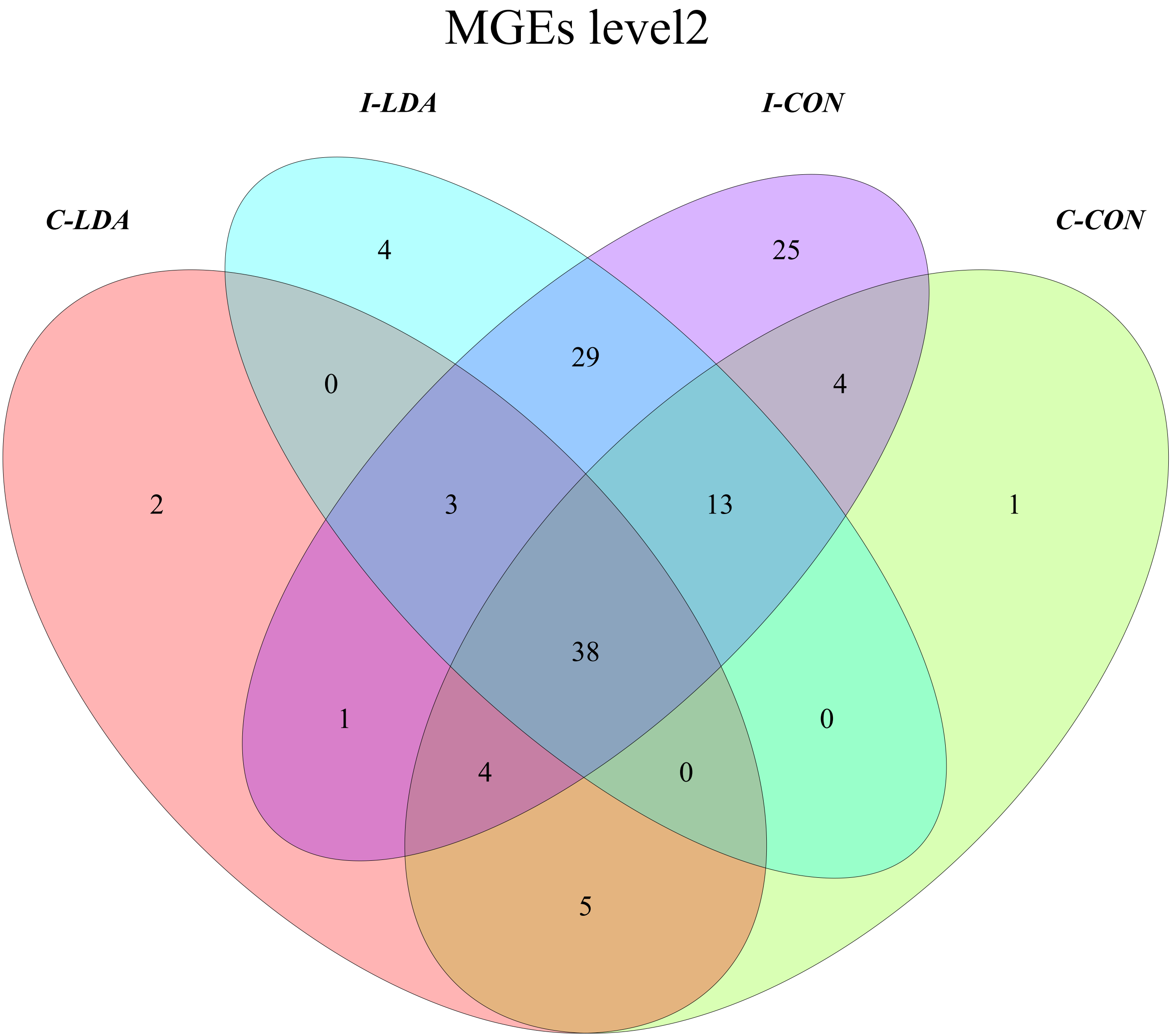

Supplementary Figure S6. Venn diagram showing distribution of the MGEs in the ileum and colon contents between LDA and CON groups.

(A)

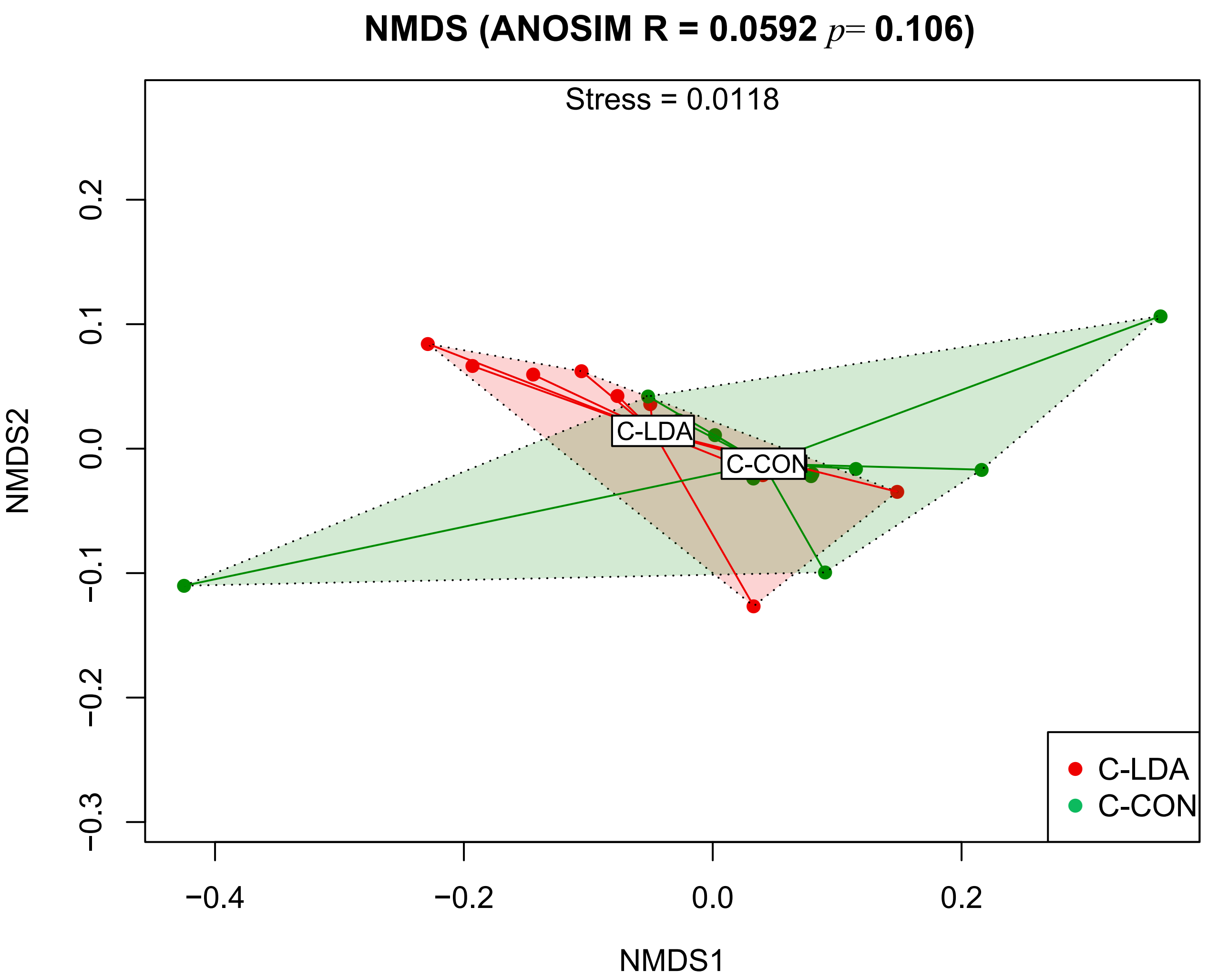

(B)

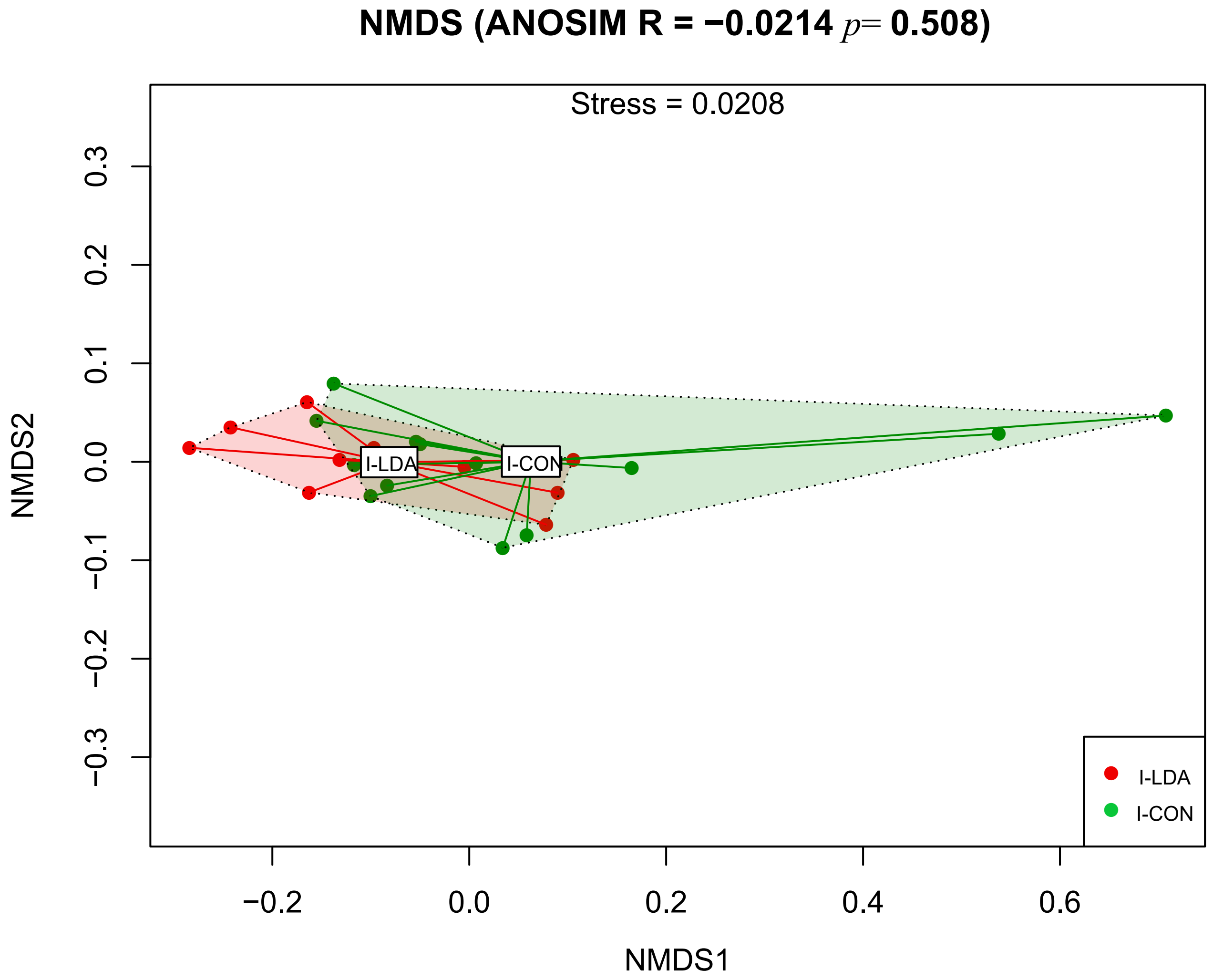

Supplementary Figure S7. Beta diversity analysis of MGEs in colon and ileum groups respectively using NMDS analysis based on Bray-Curtis distance of Features. Analysis of Similarity (ANOSIM) and effect size was indicated by an R-value (between −1 and +l, with a value of 0 representing the null hypothesis). (A) Beta diversity analysis of MGEs in colon samples. (B) Beta diversity analysis of MGEs in ileum samples.

(A)

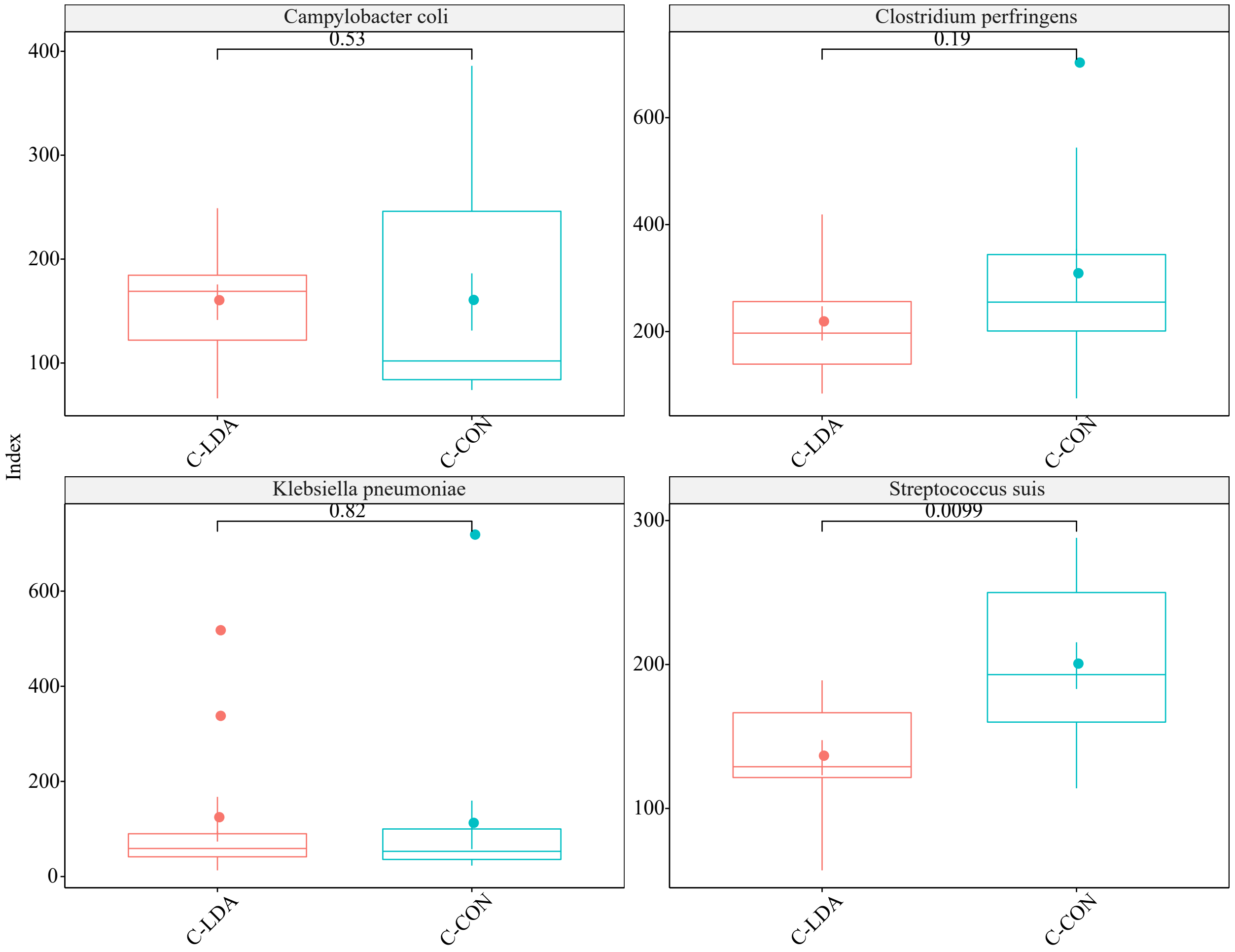

(B)

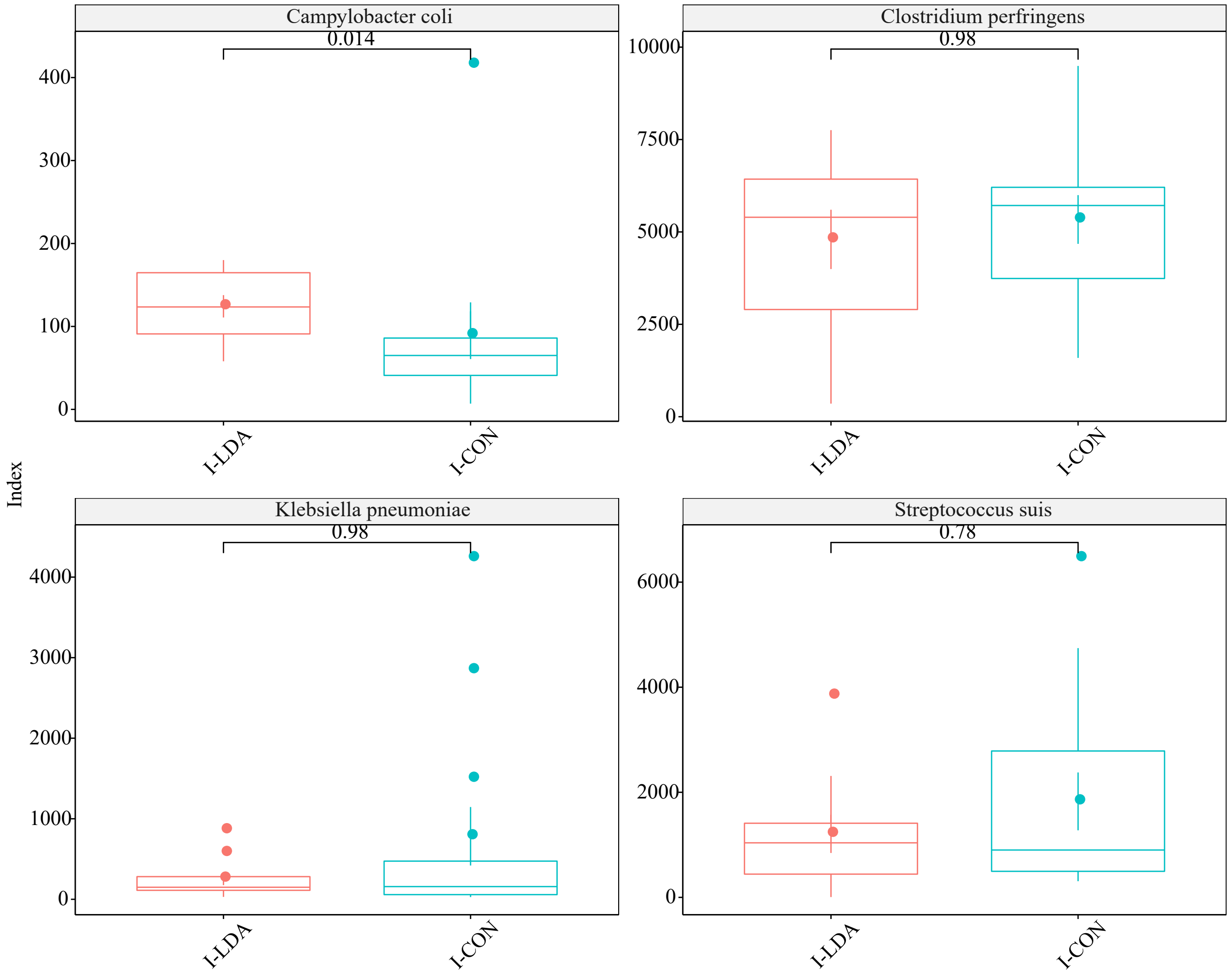

Supplementary Figure S8. The differential analysis of pathogenic bacteria species in (A) colon and (B)ileum samples between LDA and CON groups.
